# Motivations and Psychological Characteristics of Men Seeking Penile Girth Augmentation

**DOI:** 10.1093/asj/sjac112

**Published:** 2022-05-02

**Authors:** Gemma Sharp, Anne Nileshni Fernando, Michael Kyron, Jayson Oates, Peter McEvoy

**Affiliations:** Monash Alfred Psychiatry Research Centre at Monash University, Melbourne, Victoria, Australia; Monash Alfred Psychiatry Research Centre at Monash University, Melbourne, Victoria, Australia; School of Population Health and enAble Institute at Curtin University, Bentley, Western Australia, Australia; School of Population Health and enAble Institute at Curtin University, Bentley, Western Australia, Australia

## Abstract

**Background:**

The popularity of penile augmentation procedures is increasing, but little is known about the motivations and psychological characteristics of men who seek these procedures.

**Objectives:**

Employing valid psychological measures, the authors sought to investigate the motivations and psychological characteristics of men seeking penile girth augmentation.

**Methods:**

Men seeking to undergo a penile girth augmentation (n = 37) completed an online questionnaire containing standardized measures assessing their motivations to undergo augmentation, penile size self-discrepancy, psychological distress, self-esteem, body image–related quality of life, body dysmorphic disorder (BDD), and cosmetic procedure screening scale-penile focused dysmorphic disorder.

**Results:**

Men’s motivations for seeking penile girth augmentation were characterized as “improve self-confidence,” “change penile size/appearance,” “sexual function/pleasure,” “feelings of insecurity,” and “medical issues,” with self-confidence being the most commonly reported motivation. The men perceived their actual penis size (girth, flaccid length, erect length) as significantly smaller than ideal size, the size they believed their penis should be, and their expected size postaugmentation. Compared with non-clinical norms, the men seeking penile augmentation had higher penile dysmorphic disorder symptoms, lower self-esteem and lower body image–related quality of life, but comparable psychological distress. In addition, 4 of the men met diagnostic criteria for BDD according to self-reported questionnaire (11%, n = 4/37) and clinical interview (14%, n = 4/29).

**Conclusions:**

Men seek penile girth augmentation for a variety of reasons and perceive all their penile dimensions to be smaller than ideal sizes. They differ from non-clinical samples in some psychological characteristics, and a small but sizeable portion experience BDD.

See the Commentary on this article here.

A substantial proportion of men are concerned about their penis size.^1^ One large study surveying 25,592 men found that 45% of these men wanted a larger penis size compared with only 38% of men who wanted to be taller in height.^2^ Other research has shown that perceived penis size was linked to higher incidences of lying about penis size, which, in turn, was related to higher rates of penile appearance dissatisfaction.^3^ It makes sense that men who perceive their penis size to be inadequate are dissatisfied with their penile appearance and can experience poorer overall self-perception, including in their intimate relationships.^4^

Dissatisfaction with penis size has become a leading source of motivation for men to pursue penile augmentation procedures to ultimately increase the length and/or girth of their penis.^4^ Although the rate of uptake of such procedures is rarely reported in the literature, anecdotally, there have been reports of an increasing number of procedures performed by medical professionals.^4^ Notably, research has found that most men seeking penile augmentation surgeries have penis sizes that are within normal size ranges, potentially highlighting overestimation of “normal” penis size.^2,3,5,6^ The estimates provided by 87 patients (presenting at a clinic with concerns around small penis size) for what they perceive to be a “normal” flaccid penis length was 13 cm.^7^ This was higher than the average flaccid length range of 8 to 10 cm as determined by multiple population-based studies.^7^ It is possible that this lack of awareness of a range of “normal” penile appearances, in conjunction with an exaggerated presentation in media and popular culture, contributes to developing dissatisfaction with one’s own penile appearance and ultimately seeking augmentation procedures.

Research studies reporting in-depth motivations for penile augmentation directly from men themselves (as opposed to from clinician notes) are still rare. Most investigations simply state men are seeking to increase their penis size, which is, of course, a given with men presenting for augmentation procedures. Of the limited number of studies that have investigated motivations from men themselves, improving self-perception has been the most commonly reported reason.^1,5^ However, these studies were retrospective in nature and so relied on patient recall of their motivations prior to their procedure, which is a sub-optimal research design due to potential memory and social desirability biases.

As previous research investigating other aesthetic procedures has shown, in addition to examining motivations, it is also important to assess preprocedure patient expectations and psychological functioning.^8-10^ This allows clinicians to screen for patient suitability and ultimately attempt to optimize patient postprocedure satisfaction.^11,12^ The literature reports varying levels of satisfaction for different types of penile augmentation procedures; however, these data mostly have been collected in a retrospective fashion and employing single-item, non-validated measures.^3,5^ Although some studies have shown that these procedures can relieve some men of the dissatisfaction and anxiety associated with the perception of their penis size, there is a population of men whose dissatisfaction is more excessive and whose expectations are harder to meet.^3^ Body dysmorphic disorder (BDD) involves an excessive pre-occupation with a perceived defect in addition to compulsive behaviors and obsessive thoughts about the defect.^10^ The proportion of patients who seek aesthetic procedures (across types) and meet the criteria for BDD has remained approximately 5% to 15% over the last 20 years or so.^10^ Research has shown that most of the patients diagnosed with BDD do not experience the same or similar levels of satisfaction after aesthetic procedures. Some even experience a worsening of symptoms, which has therefore implied that BDD is a contraindication for aesthetic procedures.^4^ For men seeking penile girth augmentation, a BDD prevalence rate of 8% was previously reported; however, this was obtained retrospectively (ie, asking men to recall their distress prior to having the procedure), and no clinical interview was administered that is considered best practice.^5,13,14^ Thus, this 8% estimate has significant limitations, and a thorough psychological profile, including BDD, is yet to be obtained prospectively for men seeking penile girth augmentation.

In sum, in the current study, the motivations and psychological characteristics of men seeking a penile girth augmentation were examined employing a prospective design. In particular, the study aimed to determine a more comprehensive psychological profile via a series of validated measures, particularly, penile size self-discrepancy, psychological distress, self-esteem, body image–related quality of life, body dysmorphic disorder, and body dysmorphia relating to penis size.

## METHODS

### Participants

This prospective study recruited men from 3 private aesthetic surgery clinics in Australia who self-referred seeking medical penile girth augmentation (as outlined in Oates and Sharp) and who met the following inclusion criteria: 18 years or older, no local infection, no permanent filler/implant, and realistic expectations based on clinical interview.^4^ Patients did not necessarily need to proceed with the procedure to participate in our study. Note that all men involved in the study had a penile girth size within normal size ranges as measured by the medical practitioner at the initial consultation.^7^

### Measures

A questionnaire was developed by the researchers and consisted of established and validated psychological measures. A blank copy of the questionnaire is available exclusively online at www.aestheticsurgeryjournal.com as supplemental material (Appendix). The questionnaire contained the measures outlined below.

### Demographic Variables

Questions were administered assessing demographic characteristics (age, marital status, sexual orientation, ethnicity, education, and employment).

### Motivations for Penile Girth Augmentation

Participants were asked about their motivations to pursue a penile augmentation procedure in an open-ended response format to allow participants to include as many thoughts as they deemed relevant. Responses to the open-ended question of “What are your reasons for considering a penile augmentation procedure?” were read and coded for themes by the first 2 authors independently of each other.^15^ The 5 themes agreed on after discussion were “improve confidence,” “change penile size/appearance,” “sexual function/pleasure,” “feelings of insecurity,” and “medical issues.”

### Self-Discrepancy Questionnaire

The self-discrepancy questionnaire includes a series of questions regarding a participants’ estimate of the size (for both length and girth) of their flaccid and erect penis in relation to other men for (1) self-actual (what they believe their actual size is in relation to others); (2) self-ideal (how they would ideally like their penis to be in relation to others); and (3) self-should (what their penis should be in relation to others).^16^ Participants were also asked to rate expected postaugmentation outcomes in line with the self-actual discrepancy questionnaire item above (ie, what they believe their postaugmentation size will be in relation to others).

### Kessler Psychological Distress Scale

The Kessler Psychological Distress Scale (K10) is a 10-item self-report measure intended to yield a global measure of non-specific psychological distress.^17^ Typically represented by a total score with excellent internal consistency (Cronbach’s α = 0.93), the K10 discriminates individuals with and without mental disorders and has demonstrated reliability and validity across a range of populations.^17-21^ Symptoms are assessed over the prior 4 weeks on a Likert-type scale, from none of the time (1), a little of the time (2), some of the time (3), most of the time (4), and all of the time (5). Internal consistency was high in this study (Cronbach’s α = 0.91).

### Rosenberg Self-Esteem Scale

The Rosenberg Self-Esteem Scale (RSES) is a well-validated 10-item measure of global self-esteem.^22^ Items are rated on a 4-point scale from strongly disagree (1) to strongly agree (4). Five of the items are negatively worded and 5 are positively worded. The scale has demonstrated good test-retest reliability (0.82-0.88) and adequate internal consistency (Cronbach’s α = 0.77-0.88).^23,24^ There is evidence that the RSES is unidimensional in nature and is typically represented by a total score.^25^ Internal consistency in the current study was high (Cronbach’s α = 0.90).

### Body Image Quality of Life Inventory

The Body Image Quality of Life Inventory (BIQLI) is a 19-item self-report scale measuring the impact of body image concerns on a broad range of life domains (e.g., social functioning, sexuality, emotional well-being).^26^ Items are rated on a 7-point scale indicating the impact on one’s life: very positive effect (+3), no impact (0), to very negative effect (−3). The measure has demonstrated very high internal consistency (Cronbach’s α = 0.95)^26^ and good test-retest reliability over a 2- to 3-week period (r = 0.79). There is evidence that the BIQLI is unidimensional and therefore can be represented by a total score.^27,28^ Internal consistency was very high in the current study (α = 0.98). The BIQLI is calculated as a mean score across the 19 items, with more negative scores reflecting a more negative body image.

### Body Dysmorphic Disorder Questionnaire

The Body Dysmorphic Disorder Questionnaire (BDDQ) is a brief self-report screening measure for BDD based on Diagnostic and Statistical Manual of Mental Disorders (5th ed.).^29,30^ Questions assess appearance concerns and preoccupation, impacts of the preoccupation on the person’s life, and the duration of preoccupation each day. Item 2 also assesses whether the main appearance concern is that the person is not thin enough or that they might become too fat to rule out an eating disorder rather than BDD (“eating disorder exclusion”). The BDDQ has demonstrated high sensitivity (100%) and a specificity of 92.3% on a cosmetic procedure–seeking sample.^31^

### Cosmetic Procedure Screening Scale for Penile Dysmorphic Disorder

The Cosmetic Procedure Screening Scale for Penile Dysmorphic Disorder (COPS-P) is a 9-item scale for assessing perceptions and concerns around penis size and appearance.^32^ Due to an administrative error, only the first 6 items were administered. These items assessed the degree to which the individual feels the size or appearance of his penis is defective or unattractive and the extent to which the size or appearance causes distress, avoidance, preoccupation, expected impacts on sexual relationships, and interference in ability to work. Veale et al provided item-level norms for the COPS-P, which were utilized for comparison in this study.^32^ Veale et al also found a high internal consistency for the full scale (Cronbach’s α = 0.94), and the 6 items included in this study also had high internal consistency (Cronbach’s α = 0.92).^32^

### MINI International Neuropsychiatric Interview 7.0.2 Body Dysmorphic Disorder Module

The MINI is a structured diagnostic interview based on the Diagnostic and Statistical Manual of Mental Disorders (5th ed).^30,33^ The BDD module of the interview asks whether the person spends a lot of time thinking about a defect or flaw in their appearance, excessive worry, has recurrent thoughts comparing themself with others or repetitive behaviors (eg, checking), and whether these thoughts cause significant distress in important life domains. Affirmative responses to all these questions are consistent with a BDD diagnosis.

### Procedure

From July 2018 to June 2021, patients who self-referred to 1 of 3 private aesthetic surgery clinics in Australia for penile girth augmentation completed an initial assessment with a medical practitioner. Following the assessment, clinic reception staff provided patients who met the inclusion criteria with a brief information sheet and consent form to be contacted by the research team about a study designed to evaluate attitudes towards the penile augmentation procedure they were considering. Patients were informed that there was no commitment to participate if they signed this consent form. Patients who completed the written consent form were contacted by research staff independent of the clinic to provide more detailed information in verbal and written formats and another written consent form to participate in the study itself.

Consenting patients were assigned a unique study identification number and were sent a link to an online survey containing the baseline measures. The survey included demographic details and motivations, self-discrepancy questionnaire, K10, RSES, BIQLI, BDDQ, and COPS-P. Once the online measures were completed, the study research assistant arranged a time to contact the participant to complete the BDD module of the Mini International Neuropsychiatric Interview over the phone. Once completed, participants were thanked for their time. IRB approval for this study was obtained from Curtin University, Perth, Australia (HRE2018-0268).

### Analytical Procedure

The data were analyzed utilizing IBM SPSS (version 27.0; IBM SPSS, Inc, Chicago, IL). Sample characteristics are reported descriptively. Analyses examined differences in perception of actual penis size (erect length, non-erect length, girth) and what an individual believes their penis size should be, their ideal size, and what they expect their size will be postprocedure. Cohen’s d statistics were calculated to examine the magnitude of these differences.^34^

Independent samples *t* tests were then conducted to examine differences between the current sample and non-clinical populations on measures of distress (K10), self-esteem (RSES), and body image quality of life (BIQLI). Normative values were derived from Slade et al (K10), Schmitt and Allik (RSES), Cash et al (BIQLI), and Veale et al (COPS-P).^25,27,32,35,36^ To examine differences in aspects of penile dysmorphic disorder (PDD) between the current sample and normative values (derived from Veale et al), independent samples *t* tests were then conducted for the COPS-P items.^32^

To control for the increased chance of family-wise error, a Benjamini–Hochberg correction was applied to provide a more conservative rejection criterion. Specifically, the correction involves correcting the rejection criteria (ie, *P* > 0.05) by the number of tests performed. To minimize the chance of type II error, this method involves rank ordering the *P* values of all tests performed. Next, the rejection criterion is adjusted by N − 1 tests until a non-significant result is achieved.

## RESULTS

### Sample Characteristics

A total of 38 male patients commenced the online questionnaire between July 5, 2018, and March 11, 2021, with 1 not providing consent. The remaining 37 patients represent a 47% recruitment rate from those who consented to being contacted by the research team (n = 81). The men ranged from 21 to 68 years of age (mean = 40.22, standard deviation = 11.89). Most patients were single or married and heterosexual (Table 1). The sample predominantly self-reported low or mild psychological distress (n = 31/36, 86%).

**Table 1.** Sample Characteristics of Men Seeking Penile Girth Augmentation (n* *= 37)

Characteristic	Level	No. (%)
Age	Mean (SD), range	40.22 (11.89), 21-68
Marital status, no. (%)	Single	15 (39%)
	Married	11 (29%)
	In a relationship	3 (8%)
	Divorced	2 (5%)
	Widowed	1 (3%)
	Separated	5 (13%)
Sexual orientation	Heterosexual	30 (79%)
	Homosexual	3 (8%)
	Bisexual	2 (5%)
	Prefer not to say	2 (5%)
Ethnicity^a^	Australian	25 (68%)
	United Kingdom (English, Irish, Scottish)	11 (30%)
	Southern European (Italian/Greek/French)	7 (19%)
	Asian (Chinese, Indian, Indonesian)	3 (8%)
	Brazilian	1 (3%)
	New Zealand	1 (3%)
Highest level of education	Primary school	1 (3%)
	High school (up to year 10)	6 (16%)
	High school completed	6 (16%)
	TAFE/technical college	5 (14%)
	Apprenticeship	7 (19%)
	University undergraduate degree	9 (24%)
	University postgraduate degree (coursework/research)	2 (5%)
	University doctoral degree	1 (3%)
Work status	Not working	2 (5%)
	Part-time work (15-34 h/wk)	2 (5%)
	Full-time work	31 (84%)
	On temporary leave	1 (3%)
	In training (apprentice or student)	1 (3%)
Psychological distress (K10)^b^	Low	21 (57%)
	Moderate	10 (27%)
	High	3 (8%)
	Very high	2 (5%)
	Missing	1 (3%)
BDD from BDDQ	No	27 (75%)
	Yes (without presence of eating disorder)	9 (24%)
	Yes (after eating disorder exclusion)	4 (11%)
	Insufficient questionnaires completed	1 (3%)
BDD from MINI (total n = 29)	No	25 (86%)
	Yes	4 (14%)

BDD, body dysmorphic disorder; BDDQ, body dysmorphic disorder questionnaire; K10, Kessler 10-Item Distress Scale; MINI, mini international neuropsychiatric interview; SD, standard deviation; TAFE, Technical and Further Education.

^a^Multiple options could be selected so total number > 37.

^b^K10 categories are consistent with those reported in Slade et al

^35^^,^

^36^: 10-15 (low), 16-21 (moderate), 22-30 (high), 31-50 (very high).

As also seen in Table 1, in total, 9 patients met the criteria for BDD based on the BDDQ when the Item 2 eating disorder exclusion was not applied (“Is your main concern with your appearance that you aren’t thin enough or that you might become too fat?”: 24%), with only 4 patients (11%) meeting criteria when this criterion was applied. However, 1 of these 9 did not provide all self-report data so was excluded from some analyses. On the MINI BDD module, 4 patients out of the 29 (14%) who could be contacted by phone to complete the interview met the criteria, but only 1 of these 4 also met criteria on the BDDQ.

### Motivations

The participants provided a range of reasons for seeking penile girth augmentation. As seen in Table 2, the most common motivation (and most common sole motivation) was to improve self-confidence as reported by almost one-half of the participants. The next most common reasons were to change the size/appearance of their penis, sexual reasons (functional/pleasure in nature), addressing feelings of insecurity, and, finally, treating a medical issue.

**Table 2.** Motivations for Penile Augmentation by Theme (n = 36)

Theme	Example	No. (%)^a^	No. (%) as sole reason
Improve confidence	“To improve confidence” “For my self-confidence”	17 (47)	8 (22)
Change penile size/appearance	“Increase the girth of my penis a little bit” “Mainly for aesthetic purposes”	14 (39)	5 (14)
Sexual function/pleasure	“I have a want to satisfy my partner” “Better pleasure during sex”	12 (33)	3 (8)
Feelings of insecurity	“I think I’m too small and am embarrassed sometimes” “I’ve always been shy about my size and girth . . .”	8 (22)	0 (0.0)
Medical issues	“At present, I have an issue with penile retraction . . .”	1 (3)	0 (0.0)
Combination	“Feel insecure about my size during sex and am concerned about how I look when my penis is flaccid.” “Size is currency in homosexual culture. Imagine confidence gains. Curiosity. Increased satisfaction for partner.”	17 (47)	—

^a^Percentages do not sum to 100% because participants provided motivations that were coded into multiple themes.

### Self-Discrepancy for Penis Size

Discrepancies between perceived actual erectile/non-erectile/girth and “ideal” size, what patients believe their size “should be,” and “expected” (postaugmentation) size have been reported in Table 3. On average, the participants perceived their current erect length, current non-erect length, and current girth to all be below average (<50th percentile), with flaccid length being rated as the most below average. For all 3 size dimensions, the men rated their “ideal” and “should be” size dimensions most highly above average (>50th percentile) followed by “expected,” which was still above average. There were significant differences for each comparison, indicating that, on average, patients prior to treatment perceived their actual size (flaccid length, erect length, and girth) to be significantly smaller than ideal size, should be size, and expected size.

**Table 3.** Self-Discrepancy Questionnaire Scores (Percentiles) and Tests of Differences (n = 34)

Size dimension	Outcome	Mean	SD	Min	Max	Actual vs	*P*	Cohen’s *d*
Erectile length	Actual	46.03	10.35	24	65	—	—	—
	Should	68.18	11.10	50	100	−22.15	<0.001	−2.06
	Ideal	71.18	10.99	50	100	−25.15	<0.001	−2.36
	Expected	57.79	12.76	26	100	−11.76	<0.001	−1.01
Non-erectile length	Actual	33.79	14.87	9	65	—	—	—
	Should	58.38	12.08	29	82	−24.59	<0.001	−1.82
	Ideal	65.82	12.23	40	100	−32.03	<0.001	−2.35
	Expected	55.65	15.00	10	100	−21.85	<0.001	−1.46
Girth	Actual	38.56	17.54	7	70	—	—	—
	Should	61.71	16.45	14	100	−23.15	<0.001	−1.36
	Ideal	69.18	14.98	18	100	−30.62	<0.001	−1.88
	Expected	63.00	17.98	9	99	−24.44	<0.001	−1.38

SD, standard deviation.

### Psychological Distress, Self-Esteem, and Body Image Quality of Life

Scores on psychological distress, self-esteem, and body image quality of life were compared with non-clinical norms (see Table 4). Compared with non-clinical norms, the current sample had lower self-esteem (with large effect size) and lower body image–related quality-of-life scores (with medium effect size) and comparable psychological distress.

**Table 4.** Comparisons of the Current Sample With Non-clinical Normative Data for Psychological Measures

Variable	Current sample mean (SD)	Min	Max	Normative mean (SD)	Current vs norms
Psychological distress (K10, n = 36)	16.22 (5.95)	10	35	14.0 (6.34; n = 4025)	*P* = 0.036^a^, Cohen’s *d* = 0.36
Self-esteem (RSES, n = 36)	21.33 (5.65)	10	30	31.07 (5.15; n = 201)	*P* < 0.001, Cohen’s *d* = 1.80
Body image and quality of life Inventory (BIQLI, n = 35)	0.37 (1.58)	−3.00	2.63	1.24 (0.99; n = 135)	*P* < 0.001, Cohen’s *d* = 0.68

BIQLI, Body Image Quality of Life Inventory; K10, Kessler 10-Item Distress Scale; RSES, Rosenberg Self-Esteem Scale; SD, standard deviation. K10 male norms are from Slade et al^35,36^; RSES male Australian non-clinical norms are from Schmitt and Allik^25^; BIQLI norms are from Cash et al^27^.

^a^ *P* value adjusted for multiple tests—non-significant at *P* ≥ 0.017 (0.05/3).

### Body Dysmorphia Relating to Penis Size

Scores on the 6 assessed items on the COPS-P were compared with established norms.^32^ Independent samples *t* tests revealed that the current sample had significantly higher scores on all 6 COPS-P items compared with a non-clinical sample, with large effect sizes (see Table 5). The comparisons between the current sample and the PDD sample from Veale et al revealed statistically significantly lower results for the current sample for Items 2 (“To what extent does the size or appearance of your penis currently cause you distress?”) and 4 (“To what extent does thinking about the size or appearance of your penis currently preoccupy you? That is, you think about it a lot and it is hard to stop thinking about it”), with small-medium effects and medium-large effects, respectively.^32^

**Table 5.** Comparisons Between Mean (SD) COPS-P Items for Current Sample, PDD Sample, and Non-clinical Control Norms

Item	Current sample (n = 35)	Min	Max	PDD^a^ (n = 21)	Control^a^ (n = 23)	Current vs PDD^b^	Current vs control^b^
1. To what extent do you feel the size or appearance of your penis is defective or unattractive?^c^	3.83 (2.36)	0	8	5.78 (2.31)	0.43 (0.75)	*t* = 0.94, *P* = 0.349, *d* = −0.18	*t* = −9.30, *P* < 0.001, *d* = 2.16
2. To what extent does the size or appearance of your penis currently cause you distress?	3.86 (2.67)	0	8	6.28 (1.81)	0.71 (1.77)	*t* = −2.15, *P* = 0.036, *d* = −0.45	*t* = −6.56, *P* < 0.001, *d* = 1.32
3. How often does the size or appearance of your penis currently lead you to avoid situations or activities?^c^	3.60 (2.71)	0	8	5.72 (1.93)	0.57 (1.78)	*t* = −0.47, *P* = 0.638, *d* = −0.10	*t* = −7.53, *P* < 0.001, *d* = 1.52
4. To what extent does thinking about the size or appearance of your penis currently preoccupy you? That is, you think about it a lot and it is hard to stop thinking about it.	3.26 (2.39)	0	8	5.83 (1.69)	0.24 (0.54)	*t* = −2.64, *P* = 0.011, *d* = − 0.54	*t* = −7.91, *P* < 0.001, *d* = 1.94
5. If you have a regular partner, to what extent do your concerns about the size or appearance of your penis currently have an effect on an existing sexual relationship?	3.31 (2.70)	0	8	5.50 (2.55)	0.14 (0.48)	*t* = −1.63, *P* = 0.109, *d* = −0.32	*t* = −7.31, *P* < 0.001, *d* = 1.85
6. How much do your concerns about the size or appearance of your penis currently interfere with your ability to work or study?	2.97 (2.74)	0	8	3.44 (2.48)	0 (0)	*t* = 0.73, *P* = 0.471, *d* = 0.14	*t* = -6.93, *P* < 0.001, *d* = 2.05

COPS-P, Cosmetic Procedure Screening Scale for PDD; PDD; penile dysmorphic disorder; SD, standard deviation.

^a^PDD and control norms derived from Veale et al.^32^

^b^Benjamini–Hochberg *P* value correction applied—statistically significant at *P* < 0.025.

^c^Items 1 and 3 have been reverse scored such that higher scores represent higher symptoms of PDD.

## DISCUSSION

This study appears to be the first to provide a prospective and comprehensive psychological characteristic profile of men seeking penile girth augmentation using validated measures, including arguably the most relevant psychiatric disorder in the field of aesthetic procedures: BDD. As such, the study has provided important new insights into the psychological profile of these men, which provides a crucial platform for future research. There were some notable differences in the psychological profiles between men seeking penile augmentation and non-clinical norms. In particular, the men in the study had lower self-esteem and body image–related quality of life compared with non-clinical norms but comparable psychological distress. The vast majority of the sample was categorized in the low/mild psychological distress. Furthermore, a small but sizeable minority (11%-14%) of the men met criteria for BDD.

Although the men reported having a variety of motivations for seeking a penile girth augmentation, a desire to improve their self-confidence was the most commonly reported motivation. This is consistent with previous retrospective research involving men seeking a penile girth augmentation and in keeping with previous findings that men’s self-worth can be influenced by the perceived “adequacy” of their genitals.^5,37^ Nevertheless, several other reasons were identified, including a desire to change size/appearance, sexual function/pleasure, feelings of insecurity, and medical issues. This suggests that men’s reasons for seeking penile augmentation are complex, and such complexity would be expected when penis size is linked with men’s overall feelings of self-identity and masculinity.^38^

As expected, on average, the men perceived not only their current penile girth but also their flaccid and erect penile length to be below average size. In concordance with previous research, men typically overestimate average penile size, and this has been linked with exposure to pornographic materials where male porn actors tend to have particularly large penises.^1,3^ Furthermore, the men in this study believed that their ideal penis as well as what their penis “should be” was significantly greater than than their current girth and length, thus helping to explain their desire to undergo penile augmentation. The men also expected not only their girth, but also their flaccid and erect length, to be significantly larger after the girth augmentation. Although realistic expectations were specifically examined by the medical practitioners in the initial patient consult, this could potentially suggest that the men in this study had some unrealistic expectations for their postprocedure length, which is a known “red flag” when screening patients for aesthetic procedures.^10,11,39^ Nevertheless, their expected sizes were generally lower than their ideal and “should be” sizes for girth and length, indicating that they at least did not expect a result in accordance with their ideals. It will be important to reexamine whether expectations for these men were met after they underwent penile girth augmentation, which is included in our future research. From previous studies, men who undergo penile girth augmentation are generally satisfied with their postprocedure girth and length.^5,40^

The study also examined commonly investigated psychological characteristics in the men seeking penile girth augmentation and compared them with non-clinical norms. The men were experiencing lower self-esteem and body image–related quality of life than non-clinical norms. These findings suggest that the men in the study were experiencing issues with their self-esteem and quality of life but on average were not more generally distressed than the general population. These appear to be novel findings employing an optimal prospective study design and validated measures, for men seeking a penile augmentation which was a further study strength.

Previous research investigating the psychosocial functioning of patients seeking other aesthetic procedures has produced inconsistent results. Some studies have found that patients experience significant psychopathology prior to aesthetic procedures and others have psychological functioning within normal ranges.^41-46^ The major consistent finding across aesthetic surgery types is that patients experience significant psychological distress directed toward the area of the body that is the focus of the procedure.^41^ This was also the case in the current study, with the men scoring significantly higher on all administered items of the COPS-P compared with non-clinical samples and scoring comparably with a PDD sample in almost all items. Clearly, further research and, importantly, an investigation of how these psychological measures change or not after men undergo penile girth augmentation (our future research) are needed.

The study also provides an important baseline for BDD prevalence in men seeking penile augmentation using a self-report measure in the BDDQ and clinician-administered measure in the MINI BDD module. The prevalence was similar (11%-14%) for both measures when eating disorder exclusion was applied for the BDDQ. This prevalence rate is at the higher end of the range observed with other types of aesthetic procedures, which is approximately 5% to 15%.^47^ It was an unexpected finding that only 1 of the 4 patients met BDD criteria on both measures, which requires further research. However, it is possible that the difference in style of administration (ie, self-report vs clinician delivery) may help explain the result to some extent. Both measures are brief in nature, and potentially a more in-depth structured clinical interview (eg, Structured Clinical Interview for DSM Disorders) may have been beneficial to employ to investigate BDD in this sample and can be utilized in future research.^48^

Aesthetic medical practitioners are encouraged to employ their in-depth clinical interviewing skills when assessing penile augmentation patients for BDD symptoms. Practitioners could consider routinely employing a brief self-report measure such as the COPS-P with patients seeking penile augmentation prior to the initial consultation and then follow up with more in-depth questioning in the appointment with any responses of concern from the COPS-P. It may also be beneficial for surgeons to work closely with mental health professionals to assist with the psychological screening of penile augmentation patients. A patient who is deemed unsuitable by a surgeon for an augmentation procedure may, concerningly, approach non-medical professionals for unproven penile-focused augmentation procedures.^4^ Instead, a surgeon working with a mental health professional can suggest a referral for psychological input such that the patient’s concerns can still be addressed. In the first author’s extensive clinical experience, it is possible that after psychological therapy, a previously unsuitable candidate for an aesthetic procedure can be deemed suitable by the mental health professional and surgeon. For example, a patient who has highly unrealistic expectations of an aesthetic procedure for positive impacts on their intimate relationship can learn to modify these expectations to something more realistic and start to address the concerns they have about their intimate relationship in collaboration with their partner utilizing psychological strategies.

Some limitations should be considered when interpreting the results of this study. The sample size was on the smaller side, and, as such, there was not the statistical power to detect small effect sizes or conduct statistically sound subgroup analyses (eg, based on BDD diagnosis). Furthermore, the men included in this study may not have been representative of the population of men seeking penile augmentation because these patients were sourced only from private clinics in 1 country (Australia), where the clinics are known for injectable penile girth augmentations specifically. It is possible that men who seek girth augmentation are more diverse in terms of demographic characteristics than found in this study owing to a potential bias in the type of men receptive to participating in this type of study. It would have also been ideal to recruit a comparison sample of men not seeking penile augmentation matched on key demographic characteristics (eg, age) concurrently with the patient group rather than comparing with non-clinical norms. Finally, the full COPS-P measure was not administered owing to an administrative error, and so the overall scores for this measure could not be calculated and obtain a more comprehensive indication of penile focused body dysmorphia. Nevertheless, item-level norms have been established for the COPS-P, and 2 other validated measures of BDD were included.

## CONCLUSIONS

Notwithstanding these limitations, this study was able to provide novel insights into the motivations and psychological profiles of men seeking penile girth augmentation employing a prospective design and validated psychometric measures. It was found that men were most commonly seeking to improve their self-confidence through an augmentation procedure and perceived their penile girth and length size to be below average. There were also some notable differences in the psychological profiles between men seeking penile augmentation and non-clinical norms, particularly lower self-esteem and body image–related quality of life. Furthermore, BDD was present in 11% to 14% of these men. The study results will potentially assist clinicians in their preprocedural psychological screening of men seeking a penile augmentation and inform their clinical decision-making.

## Supplementary Material

sjac112_suppl_Supplementary_Data
