# Supplementary material for: Motivations and Psychological Characteristics of Men Seeking Penile Girth Augmentation
Source: Aesthet Surg J. 2022 May 2;42(11):1305–15. doi: 10.1093/asj/sjac112 (PMC9558456; doi:10.1093/asj/sjac112)
Supplement: sjac112_suppl_Supplementary_Data [file sjac112_suppl_supplementary_data.pdf]

Participant Consent Form

Declaration by Participant

By signing the consent form I declare that:
I have read the Participant Information Sheet or someone has read it to me in a language that I understand.
I understand the purposes, procedures and risks of the research described in the project.
I have had an opportunity to ask questions and I am satisfied with the answers I have received.
I freely agree to participate in this research project as described and understand that I am free to withdraw at any time during the project without affecting my relationship with the organisations involved.
I understand that I will be given a signed copy of this document to keep.
I agree to my medical information (penile measurements before and after treatment) from Academy Face and Body being released for use in this study.
I agree for my data to be retained in a restricted use data repository for 7 years at Curtin University, per the Western Australian University Sector Disposal Authority policy.

Curtin University Human Research Ethics Committee (HREC) has approved this study (HREC number HR2018-0268). Should you wish to discuss the study with someone not directly involved, in particular, any matters concerning the conduct of the study or your rights as a participant, or you wish to make a confidential complaint, you may contact the Ethics Officer on (08) 9266 9223 or the Manager, Research Integrity on (08) 9266 7093 or email hrec@curtin.edu.au.

- ◯ Yes, I consent to participate in this study
- ◯ No, I do not consent to participate

Do you consent to de-identified medical images (of your genital region, before and after treatment) to be used for this research (please tick to indicate your preference):

- ◯ Yes, I consent for my de-identified medical images to be used for research purposes
- ◯ No, I do not consent for my images to be used for research purposes

Demographics

What is your age (in years)?

Sexual orientation

- ◯ Heterosexual or straight
- ◯ Homosexual
- ◯ Bisexual
- ◯ Another sexuality (please describe)
- ◯ Prefer not to say

What is your ancestry? (provide up to 2 only)

- ☐ Australian
- ☐ English
- ☐ Irish
- ☐ Italian
- ☐ Chinese
- ☐ German

- [ ] Scottish
- [ ] Other

What is your highest level of education?

- ○ Primary school
- ○ High school (up to year 10)
- ○ High school (completed year 12)
- ○ TAFE (or similar)
- ○ Apprenticeship
- ○ University bachelor degree
- ○ University postgraduate masters (coursework or research)
- ○ University doctoral degree

Which of the following statements about occupational status apply to you?

- ○ Not working at the moment
- ○ Part-time or hourly work (< 15 hours per week)
- ○ Part-time work (15 to 34 hours per week)
- ○ Full-time work
- ○ On temporary leave
- ○ In training (apprentice or student)

Martial status

- ○ Single
- ○ Married (defacto or registered)
- ○ In a relationship (not married or defacto)
- ○ Divorced
- ○ Widowed
- ○ Separated

Gender

- ○ Male
- ○ Female
- ○ Trans-sexual
- ○ Intersex
- ○ Another gender (please specify)

Open-ended questions

What are your reasons for considering a penile augmentation procedure?

K-10

The following ten questions ask about how you have been feeling in the last four weeks. For each question, select the option that best describes the amount of time you felt that way.

In the last four weeks, about how often did you feel tired out for no good reason?

None of the time	A little of the time	Some of the time	Most of the time	All of the time
○	○	○	○	○

In the last four weeks, about how often did you feel nervous?

None of the time	A little of the time	Some of the time	Most of the time	All of the time
○	○	○	○	○

In the last four weeks, about how often did you feel so nervous that nothing could calm you down?

None of the time	A little of the time	Some of the time	Most of the time	All of the time
○	○	○	○	○

In the last four weeks, about how often did you feel hopeless?

None of the time	A little of the time	Some of the time	Most of the time	All of the time
○	○	○	○	○

In the last four weeks, about how often did you feel restless or fidgety?

None of the time	A little of the time	Some of the time	Most of the time	All of the time
○	○	○	○	○

In the last four weeks, about how often did you feel so restless you could not sit still?

None of the time	A little of the time	Some of the time	Most of the time	All of the time
○	○	○	○	○

In the last four weeks, about how often did you feel depressed?

None of the time	A little of the time	Some of the time	Most of the time	All of the time
○	○	○	○	○

In the last four weeks, about how often did you feel that everything was an effort?

None of the time	A little of the time	Some of the time	Most of the time	All of the time
○	○	○	○	○

In the last four weeks, about how often did you feel so sad that nothing could cheer you up?

None of the time	A little of the time	Some of the time	Most of the time	All of the time
○	○	○	○	○

In the last four weeks, about how often did you feel worthless?

None of the time	A little of the time	Some of the time	Most of the time	All of the time
○	○	○	○	○

RSES

Please select the appropriate answer for each statement that represents you

On the whole, I am satisfied with myself

Strongly Agree	Agree	Disagree	Strongly Disagree
○	○	○	○

At times I think I am no good at all

Strongly Agree	Agree	Disagree	Strongly Disagree
○	○	○	○

I feel that I have a number of good qualities

Strongly Agree	Agree	Disagree	Strongly Disagree
○	○	○	○

I am able to do things as well as most people

Strongly Agree	Agree	Disagree	Strongly Disagree
○	○	○	○

I feel that I do not have much to be proud of

Strongly Agree	Agree	Disagree	Strongly Disagree
○	○	○	○

I certainly feel useless at times

Strongly Agree	Agree	Disagree	Strongly Disagree
○	○	○	○

I feel that I am a person of worth, at least on an equal plane with others

Strongly Agree	Agree	Disagree	Strongly Disagree
○	○	○	○

I wish I could have more respect for myself

Strongly Agree	Agree	Disagree	Strongly Disagree
○	○	○	○

All in all, I am inclined to feel that I am a failure

Strongly Agree	Agree	Disagree	Strongly Disagree
○	○	○	○

I take a positive attitude to myself

Strongly Agree	Agree	Disagree	Strongly Disagree
○	○	○	○

BDDQ

This questionnaire assesses concerns about physical appearance. Please read each question carefully and select the answer that best describes your experience. Also write in answers where indicated.

Are you very concerned about the appearance of some part(s) of your body that you consider especially unattractive?

Yes	No
○	○

Do these concerns preoccupy you? That is, you think about them a lot and wish you could think about them less?

Yes	No
○	○

What are they? (Examples of areas of concern include: your skin (e.g., acne, scars, wrinkles, paleness, redness); hair (e.g., hair loss or thinning); the shape or size of your nose, mouth, jaw, lips, stomach, hips, etc.; or defects of your hands, genitals, breasts, or any other body part)

What specifically bothers you about the appearance of these body part(s)? (Explain in detail)

Is your main concern with your appearance that you aren't thin enough or that you might become too fat?

Yes ○ No ○

What effect has your preoccupation with your appearance had on your life

Has your defect(s) caused you a lot of distress or emotional pain?

Yes ○ No ○

Has it significantly interfered with your life?

Yes ○ No ○

How has your preoccupation with your appearance significantly interfered with your life?

Has your defect(s) significantly interfered with your school work, your job, or your ability to function in your role (e.g., as a homemaker)?

Yes ○ No ○

How has your defect(s) significantly interfered with your school work, your job, or your ability to function in your role (e.g., as a homemaker)?

Are there things you avoid because of your defect(s)?

Yes ○ No ○

What do you avoid because of your defects?

Have the lives or normal routines of your family or friends been affected by your appearance concerns?

Yes ○ No ○

How have the lives or normal routines of your family or friends been affected by your appearance concerns?

How much time do you spend thinking about your defect(s) per day on average? (add up all the time you spend) (choose one option)

- Less than 1 hour a day
- 1 - 3 hours a day
- More than 3 hours a day

BIQLI

Different people have different feelings about their physical appearance. These feelings are called body image. Some people are generally satisfied with their looks, whereas others are dissatisfied. At the same time, people differ in terms of how their body image experiences affect other aspects of their lives. Body image may have positive effects, negative effects, or no effect at all.

Listed below are various ways that your own body image may or may not influence your life. For each item, answer how and how much your feelings about your appearance affect that aspect of your life. Before answering each item, think carefully about the answer that is most accurate about how your body image usually affects you.

My basic feelings about myself - feelings of personal adequacy and self-worth

Very negative effect	Moderately negative effect	Slight negative effect	No effect	Slight positive effect	Moderate positive effect	Very positive effect
○	○	○	○	○	○	○

My feelings about my adequacy as a man or woman - feelings of masculinity or femininity

Very negative effect	Moderately negative effect	Slight negative effect	No effect	Slight positive effect	Moderate positive effect	Very positive effect
○	○	○	○	○	○	○

My interactions with people of my own sex

Very negative effect	Moderately negative effect	Slight negative effect	No effect	Slight positive effect	Moderate positive effect	Very positive effect
○	○	○	○	○	○	○

My interactions with people of the other sex

Very negative effect	Moderately negative effect	Slight negative effect	No effect	Slight positive effect	Moderate positive effect	Very positive effect
○	○	○	○	○	○	○

My experiences when I meet new people

Very negative effect	Moderately negative effect	Slight negative effect	No effect	Slight positive effect	Moderate positive effect	Very positive effect
○	○	○	○	○	○	○

My experiences at work or at school

Very negative effect	Moderately negative effect	Slight negative effect	No effect	Slight positive effect	Moderate positive effect	Very positive effect
○	○	○	○	○	○	○

My relationships with friends

Very negative effect	Moderately negative effect	Slight negative effect	No effect	Slight positive effect	Moderate positive effect	Very positive effect
○	○	○	○	○	○	○

My relationships with family members

Very negative effect	Moderately negative effect	Slight negative effect	No effect	Slight positive effect	Moderate positive effect	Very positive effect
○	○	○	○	○	○	○

My day-to-day emotions

Very negative effect	Moderately negative effect	Slight negative effect	No effect	Slight positive effect	Moderate positive effect	Very positive effect

My satisfaction with my life in general

Very negative effect	Moderately negative effect	Slight negative effect	No effect	Slight positive effect	Moderate positive effect	Very positive effect

My feelings of acceptability as a sexual partner

Very negative effect	Moderately negative effect	Slight negative effect	No effect	Slight positive effect	Moderate positive effect	Very positive effect

My enjoyment of my sex life

Very negative effect	Moderately negative effect	Slight negative effect	No effect	Slight positive effect	Moderate positive effect	Very positive effect

My ability to control what and how much I eat

Very negative effect	Moderately negative effect	Slight negative effect	No effect	Slight positive effect	Moderate positive effect	Very positive effect

My ability to control my weight

Very negative effect	Moderately negative effect	Slight negative effect	No effect	Slight positive effect	Moderate positive effect	Very positive effect

My activities for physical exercise

Very negative effect	Moderately negative effect	Slight negative effect	No effect	Slight positive effect	Moderate positive effect	Very positive effect

My willingness to do things that might call

Very negative effect	Moderately negative effect	Slight negative effect	No effect	Slight positive effect	Moderate positive effect	Very positive effect

My daily grooming activities (i.e., getting dressed and physically ready for the day)

Very negative effect	Moderately negative effect	Slight negative effect	No effect	Slight positive effect	Moderate positive effect	Very positive effect

How confident I feel in my everyday life

Very negative effect	Moderately negative effect	Slight negative effect	No effect	Slight positive effect	Moderate positive effect	Very positive effect

How happy I feel in my everyday life

Very negative effect	Moderately negative effect	Slight negative effect	No effect	Slight positive effect	Moderate positive effect	Very positive effect

COPS-P

This questionnaire is about the way you feel about the size or appearance of your penis. Please read the questions carefully and select the response which best describes the way that you feel about your penis. Please read the labels carefully to ensure you are selecting the answer that reflects how you feel, as some of the answers are worded in a reverse order. Please answer for how you feel over the past week.

To what extent do you feel the size or appearance of your penis is defective or unattractive?

0 Very defective	1	2 Markedly defective	3	4 Moderately defective	5	6 Slightly defective	7	8 Not at all
○	○	○	○	○	○	○	○	○

To what extent does the size or appearance of your penis currently cause you distress?

0 Not at all distressing	1	2 Slightly distressing	3	4 Moderately distressing	5	6 Markedly distressing	7	8 Extremely distressing
○	○	○	○	○	○	○	○	○

How often does the size or appearance of your penis currently lead you to avoid situations or activities?

0 Always avoid	1	2 Avoid about three quarters of the time	3	4 Avoid about half the time	5	6 Avoid about a quarter of the time	7	8 Never
○	○	○	○	○	○	○	○	○

To what extent does thinking about the size or appearance of your penis currently preoccupy you? That is, you think about it a lot and it is hard to stop thinking about it

0 Not at all preoccupied	1	2 Slightly preoccupied	3	4 Moderately preoccupied	5	6 Very preoccupied	7	8 Extremely preoccupied
○	○	○	○	○	○	○	○	○

If you have a regular partner, to what extent do your concerns about the size or appearance of your penis currently have an effect on an existing <u>sexual relationship</u>? (e.g. enjoyment of sex, frequency of sexual activity). If you do not have a regular partner, to what extent do your concerns about your penis currently stop you from developing a sexual relationship?

0 Not at all	1	2 Slightly	3	4 Moderately	5	6 Markedly	7	8 Extremely or avoid sex
○	○	○	○	○	○	○	○	○

How much do your concerns about the size or appearance of your penis currently interfere with your ability to work or study? (Please rate this even if you are not working or studying: we are interested in your ability to work or study)

0 Not at all	1	2 Slightly	3	4 Moderately	5	6 Markedly	7	8 Extremely or avoid sex
○	○	○	○	○	○	○	○	○

To what extent do your concerns about the size or appearance of your penis currently interfere with your social life? (with other people, e.g., going to parties, pubs, clubs, outings, visits)

0 Not at all	1	2 Slightly	3	4 Moderately	5	6 Markedly	7	8 Very severely
○	○	○	○	○	○	○	○	○

To what extend do your concerns about the size or appearance of your penis currently interfere with leisure activities? (for example being in a public changing room).

0 Not at all	1	2 Slightly	3	4 Moderately	5	6 Markedly	7	8 Very severely
○	○	○	○	○	○	○	○	○

How much do you feel the size or appearance of your penis is the most important aspect of who you are?

0 Not at all	1	2 Slightly	3	4 Moderately	5	6 Mostly	7	8 Totally
○	○	○	○	○	○	○	○	○

self-discrepancy

The scale below represents the LENGTH of a man's ERECT penis compared to that of other men. "0" represents the shortest penis length, "50" is the average penis length and "100" is the longest penis that exists. Anything below 50 is below average. Anything above 50 is above average.

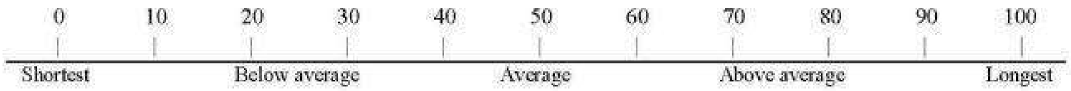

	0	10	20	30	40	50	60	70	80	90	100
Using this scale, what do you believe the **ACTUAL length of your ERECT penis is** in relation to those of other men?											
Using this scale, what do you believe the **length of your ERECT penis SHOULD be** (for whatever reason)?											
Using this scale, what **IDEALLY do you want the length of your ERECT penis to be** in relation to those of other men?											

The scale below represents the LENGTH of a man's NON-ERECT penis compared to that of other men. '0' represents the shortest penis length, '50' is the average penis length and '100' is the longest penis that exists. Anything below 50 is below average. Anything above 50 is above average.

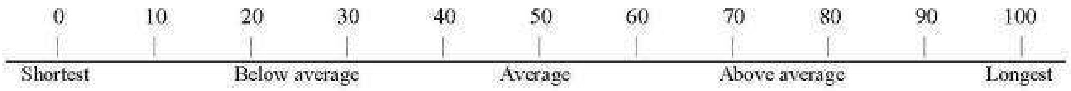

	0	10	20	30	40	50	60	70	80	90	100
Using this scale, what do you believe the **ACTUAL length of your NON-ERECT penis is** in relation to those of other men?											
Using this scale, what do you believe the **length of your NON-ERECT penis SHOULD be** (for whatever reason)?											

	0	10	20	30	40	50	60	70	80	90	100
Using this scale, what **IDEALLY do you want the length of your NON-ERECT penis to be** in relation to those of other men?											

The scale below represents the GIRTH of a man’s ERECT penis compared to that of other men. ‘0’ represents the smallest ERECT penis girth, ‘50’ is the average ERECT penis girth and ‘100’ is the largest ERECT penis girth that exists. Anything below 50 is below average. Anything above 50 is above average.

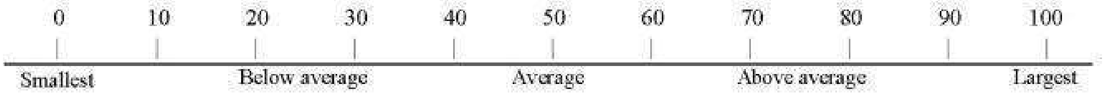

	0	10	20	30	40	50	60	70	80	90	100
Using this scale what do you believe the **ACTUAL girth of your ERECT penis is** in relation to those of other men?											
Using this scale, what do you believe the **girth of your ERECT penis SHOULD be** in relation to those of other men (for whatever reason)?											
Using this scale, what **IDEALLY do you want the girth of your ERECT penis to be** in relation to those of other men?											

self-discrepancy (expectations)

The following questions relate to HOW YOU EXPECT YOU WILL FEEL if you were to proceed with the CALIBRE treatment.

The scale below represents the LENGTH of a man’s ERECT penis compared to that of other men. "0" represents the shortest penis length, "50" is the average penis length and "100" is the longest penis that exists. Anything below 50 is below average. Anything above 50 is above average.

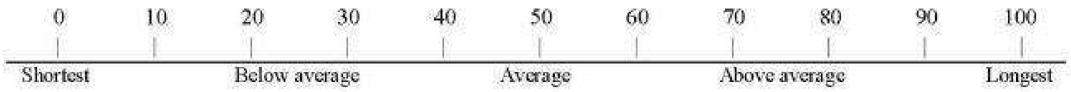

	0	10	20	30	40	50	60	70	80	90	100
	0	10	20	30	40	50	60	70	80	90	100
Following treatment, what do you believe the **<u>ACTUAL length of your ERECT penis WILL BE</u>** in relation to those of other men?											
Following treatment, what **<u>IDEALLY do you want the length of your ERECT penis to be</u>** in relation to those of other men?											

The scale below represents the LENGTH of a man's NON-ERECT penis compared to that of other men. '0' represents the shortest penis length, '50' is the average penis length and '100' is the longest penis that exists. Anything below 50 is below average. Anything above 50 is above average.

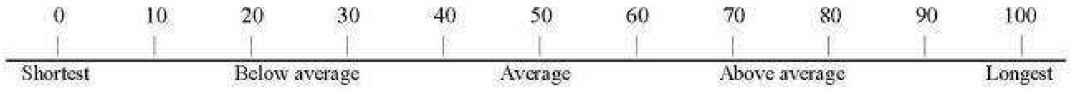

	0	10	20	30	40	50	60	70	80	90	100
Following treatment, what do you believe the **<u>ACTUAL length of your NON-ERECT penis WILL BE</u>** in relation to those of other men?											
Following treatment, what **<u>IDEALLY do you want the length of your NON-ERECT penis to be</u>** in relation to those of other men?											

The scale below represents the GIRTH of a man's ERECT penis compared to that of other men. '0' represents the smallest ERECT penis girth, '50' is the average ERECT penis girth and '100' is the largest ERECT penis girth that exists. Anything below 50 is below average. Anything above 50 is above average.

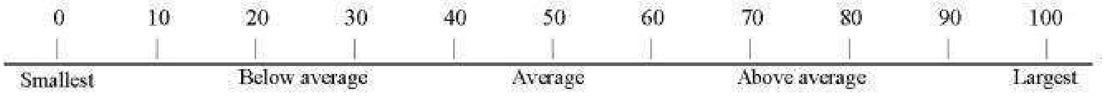

	0	10	20	30	40	50	60	70	80	90	100

	0	10	20	30	40	50	60	70	80	90	100
Following treatment, what do you believe the **ACTUAL girth of your ERECT penis WILL BE** in relation to those of other men?											
Following treatment, what **IDEALLY do you want the girth of your ERECT penis to be** in relation to those of other men?											

Thank you

Thank you very much for completing this survey. Our research assistant will contact you shortly to complete a brief (~10 minute) phone interview. After this you have completed your participation in this phase of the study. We will contact you again in 6 months to complete the final follow-up.

If you have any concerns or are distressed by any of the questions, please feel free to contact your cosmetic surgeon, your general practitioner, or any of the services listed at this link: Support services

Please click the arrow below to complete this survey.
